# The development of the asymmetrically dominated decoy effect in young children

**DOI:** 10.1038/srep22678

**Published:** 2016-03-03

**Authors:** Shanshan Zhen, Rongjun Yu

**Affiliations:** 1 School of Psychology and Center for Studies of Psychological Application, South China Normal University, Guangzhou, China; 2 Department of Psychology, National University of Singapore, Singapore, Singapore; 3 Singapore Institute for Neurotechnology (SINAPSE), Centre for Life Sciences, National University of Singapore, Singapore, Singapore; 4 Neurobiology/Aging programme, Center for Life Sciences, National University of Singapore, Singapore, Singapore

## Abstract

One classic example of context-independent violations is the asymmetrically dominated decoy effect, in which adding a decoy option (inferior option) to a set of original options often increases the individual’s preference for one option over the other original option. Despite the prevalence of this effect, little is known about its developmental origins. Moreover, it remains contentious whether the decoy effect is a result of biological evolution or is learned from social experience. Here, we investigated the decoy effect in 3- to 7-year-old children (n = 175) and young adults (n = 52) using a simple perceptual task. Results showed that older children (5-year-olds and 7-year-olds), but not younger children (3-year-olds), exhibited a decoy effect. Nevertheless, children as young as age 5 exhibited a decoy effect that was not significantly different from that shown by young adults. These findings suggest that humans start to appreciate the relative values of options at around age 5.

Rational decision making theories assume that decision makers are context-independent in decision making, meaning that decisions are made independent of irrelevant options^1^. However, decision contexts provide essential cues for people to make decisions in an uncertain world. Contextual information can bias decision making in certain situations, but can also guide individuals to make quick and sound choices, especially in uncertain situations^2^,^3^,^4^,^5^. For example, someone may vacillate between two cars (e.g., A is inexpensive but poor quality and B is higher quality but expensive), because A and B are competitive with each other on two attributes of differing dimensions (i.e., economy and quality). When adding a third car to the choice set, which is similar to A but poorer than A on quality, it will increase the individual’s preference for A and finally lead individuals to make a decision.

This phenomenon of context-dependent preference is called asymmetrically dominated decoy effect^6^. According to the decoy effect, the addition of a third option (decoy option) increases the probability of choosing a similar but superior option from among the original choice set (target vs. competitor option), although individuals theoretically should be indifferent to the presence of this third option^6^. The decoy effect violates the principle of irrelevant alternatives, which assumes that preference between options does not depend on the presence or absence of other options^7^. The prevalence of the decoy effect suggests that humans use comparative evaluation mechanisms rather than absolute preferences to make decisions^8^.

The asymmetrically dominated decoy effect is a robust context-dependent preference in the case of multi-alternative choice^6^,^9^,^10^,^11^,^12^,^13^,^14^. The dominance heuristic theory suggests that the dominating relation of the target to the decoy option provides an easily available justification for choosing the target^2^,^15^,^16^ (e.g., the target option dominates the decoy, but the competitor alternative does not; thus the target become the superior choice). Despite the prevalence of the decoy effect, little is known about its developmental origins. It remains unknown whether the decoy effect is evolutionarily adaptive and is thus hard-wired in the human brain (i.e., biological evolution), or a result of learning from social experience (i.e., cultural evolution).

Examining the decoy effect in young children has important implications for understanding the source of the decoy effect and other examples of humans’ irrational decisions. Furthermore, the decoy effect has been shown in other species, such as hummingbirds^17^,^18^, jays and bees^19^,^20^, and rhesus macaques^21^. It has been argued that such decoy effect heuristics could be favored by natural selection because they are computationally more efficient^18^,^21^. For example, using a perceptual size-discrimination task, a recent study with rhesus macaques found that a decoy stimulus impacted monkeys’ perceptual choice behavior even when it was not a viable choice option itself^21^. The findings that nonhuman animals exhibited perceptual decoy effects support the possibility that the decoy effect may emerge in young children who do not have much decision making experience yet. This study also suggests that perceptual tasks of this nature are accessible to many species and populations (e.g. young children) since they require little training or verbal instruction that might impact decision-making strategies.

According to these findings, when children compare other options with the decoy option, they may depend more on the dominance heuristic option (i.e., asymmetrically dominated by a target option) to solve the problem. One classic model of the decoy effect called the value-shift model suggests that the decoy (inferior option) enhances the dimensional value of the target on one attribute dimension relative to the competitor through processes that produce contrast effects in dimensional judgment^10^,^11^,^16^,^22^,^23^, which is consistent with the comparative evaluation mechanisms. Moreover, compared with adults, children have weaker cognitive ability and may tend to rely more on evolutionally wired heuristic processing. If so, the decoy effect should be present early in development, even in very young children.

Here, to investigate the developmental trend of the decoy effect, we adapted a simple perceptual decision task that only involved object size comparisons, which is similar to the task used by Trueblood *et al.* with adults and Parrish *et al.* with Rhesus macaques^14^,^21^. Studies on the decoy effect in adults often use complicated decision problems, including consumer decisions^24^ and decisions about jobs^2^ and political candidates^25^. Such designs are unsuitable for children, who may lack the necessary social experience to understand the questions and have little motivation to be engaged in these tasks. In our perceptual task, participating children were told to select the rectangle that had the largest area on each trial. This paradigm is culture-free and can be used across ages. Because children as young as 3 years old show functional comparisons of the size of objects^26^, the perceptual task should impose no difficulty to children at and above the age of 3. Thus, in order to examine developmental origins behind the context-dependent preference in decision making, we concentrated on the decoy effect in preschoolers and school-age children (3-to 7-year-old children) in this study, and compared their decoy effect with young adults so as to further investigate the developmental trend of the decoy effect.

## Method

### Participants

A total of 227 subjects (52 young adults, 175 children) participated in the study. Based on different grade levels and biological age, children were divided into three groups: 58 three-year-olds (mean: 3;7 years, range: 3;3–4;3, 34 female); 60 five-year-olds (mean: 5;7 years, range: 5;0–6;7, 17 female); and 57 seven-year-olds (mean: 7;8 years, range: 7;0–8;9, 23 female). The adult sample consisted of 52 young adults (25 male; mean age ± SD, 20.60 ± 1.56 years) recruited from South China Normal University. All children were recruited from a public elementary school and day-care kindergarten in the region (3- to 5-year-old children attended the same kindergarten). All children in the school were encouraged to participate. Ethnicity information was collected via parental report and with help from schoolteachers. Although socioeconomic background information was not collected for individual participants, the schools served lower to upper middle class families. All participants were identified as Han Chinese. The study was approved by the Ethics Committee of the Department of Psychology at South China Normal University. The methods were carried out in accordance with the approved guidelines. Written, informed consent was obtained from the parents of all children and young adults who participated in this study. All participants were informed of their right to discontinue participation at any time.

### Experimental paradigm

**Figure 1:**
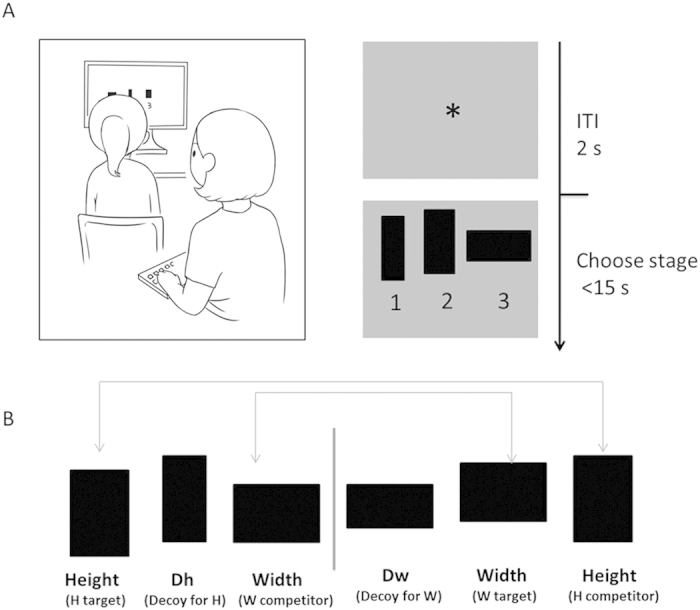
(**A**) Experimental task design. In game trials, an asterisk was on the screen for 2 s to engage attention and eye fixation at the beginning of each trial. Then three options were presented for 15 s, during which participants were instructed to make a choice by telling the experimenter the number (for the child group) that appeared beneath the chosen option. The experimenter would press the key on a computer to record the child’s choice. (**B**) Example of two decoy trials. W and H represented two choice options (width target and height target options) on a given trial, with Dw and Dh representing decoy options for W and H, respectively. The left panel showed a trial in which H was the target option, and the right panel showed a trial in which W was the target option.

Participants sat in front of a computer in a quiet room. They were then told that they would see three rectangles on each trial, and they were asked to select the rectangle that had the largest area. Three numbers (i.e., 1, 2, or 3) appeared concurrently underneath these rectangles. For the child group, participants selected the option by telling the number to the experimenter (Fig. 1A); the young adult group instead pressed the appropriate key on a computer by themselves. Participants were also told that during the task they would not receive feedback concerning their performance. The experiment session lasted for 10 min and there was one short break during the experiment.

Before beginning the game, we asked children comprehension questions to ensure that they understood the tasks (pretest). First, we showed children three real rectangles with different sizes and asked children to point to the biggest one. Children’s ability to judge the difference between the rectangle sizes in the absence of a decoy option was measured during these pretest trials. This procedure was repeated 3 times and the responses were noted. All subjects answered all questions correctly either spontaneously or after additional prompts (5% of 3-year-old children did not answer all questions correctly at first, but did so after additional questioning). Second, children had to repeat the rules of the game in their own words after the explanation by the experimenter. All children repeated the rules accurately, with the exception of two children who repeated the rules correctly after the experimenter repeated the instruction once more. Thus, all children participated in the game after the pretest phase. Finally, to increase children’s interest in participating, three kinds of snacks (i.e., yogurt, cookie, chocolate) were shown and children were told that they could get their favorite snack after they completed the game carefully (note that all children regardless of performance eventually received a prize). Similarly, young adults received a base payment (¥10, about $1.5) and extra earnings (¥5~10), depending on their performance in the task.

On each trial, three rectangles were presented on the screen from left to right. The rectangles were solid black and the background screen was grey. The vertical placements of the rectangles varied so that they did not all sit on the same horizontal axis. The rectangles were numbered from left to right (i.e., 1, 2, 3), and the rectangles (i.e., decoy, target and competitor) were presented in three fixed locations (left, center and right) randomly across trials. Three options were presented simultaneously for 15 s (including the reaction period, during which the experimenter helped children to press the key according to their choice). The experimenter repeated each verbal choice in a monotonous and controlled voice before recording the choice. If the child experienced an incongruence, s(he) was instructed to make a quick correction, e.g., say “No, not three, two”. Only in rare cases (<0.5%) were such corrections were required. This time limit permitted enough response time for children and young adults to make a decision and also created a sense of time pressure, which was important for promoting intuitive processing.

We used ternary choice sets to test the decoy effect when the height of the target option was greater than width (H target) and other ternary choice sets when the width of the target option was greater than height (W target) (Fig. 1B). Counterbalancing the stimuli in this way avoided confounding the decoy effect with preference for one type of rectangle (e.g., preference for a wide rectangle over a tall rectangle). In this case, we could compare the choice proportion of the Width target with the Width competitor and the Height target with the Height competitor across all trials and calculate the decoy effect across all trials for each subject (i.e., choice proportion for the target – choice proportion for the competitor; see Results).

These two choice sets for each target led to two types of decoy option (i.e., decoy for W: Dw; decoy for H: Dh) (also see Fig. 1B). Each participant completed 36 randomized trials, 18 with choice sets in which the height was greater than width (H target), 18 with choice sets in which the width was greater than height (W target), and 12 “catch” trials. “Catch” trials were included to assess participants’ accuracy and ensure that they remained actively engaged in the decision making task throughout the experiment. “Catch” trials also used ternary choice sets and always contained one alternative that clearly had a larger area than the rest, which provided participants with an objectively correct option.

All stimuli were displayed on a computer screen, and the monitor was set to a resolution of 1024 × 768 pixels. The height and width of each rectangle were specified in pixels (see Table 1). The mean height of W was 46 pixels and the mean width of W was 82 pixels. The variance in each dimension was 5 pixels, with no correlation between the variance in height and width. The height and width of H were edited to the same size as W. That is, the mean height of H was 82 pixels, and the mean width of H was 46 pixels. On each trial, H and W were matched so that they had the same area. The decoy option was a little weaker than the target alternative on the target alternative’s weakest attribute. Thus, the target stimulus was the rectangle enhanced by the decoy stimulus. There were two sizes for each type of the decoy option, so that the 36 trials included 3 width pixels ×3 height pixels ×2 attribute dimensions ×2 decoy sizes.

## Results

Fourteen children’s data were removed from the analysis because their accuracy on catch trials was less than threshold (60%) after the pretest phase (3-year-olds: 4 children, 2 female; 5-year-olds: 6 children, 3 female; 7-year-olds: 4 children, 2 female), leaving a final sample of n = 161 for analysis. The proportion of correct choice in the remaining children was markedly high among all three groups (3-year-olds: 0.895 ± 0.014; 5-year-olds: 0.949 ± 0.011; 7-year-olds: 0.962 ± 0.008, mean ± SE) in catch trials, providing evidence that children were able to carry out size comparisons and maintain their attention on the task. In addition, in all three groups, the decoy options were rarely chosen (3-year-olds: 0.021 ± 0.005, 5-year-olds: 0.013 ± 0.004, 7-year-olds: 0.005 ± 0.002). In addition, the young adults’ accuracy (0.990 ± 0.004) on catch trials was high and the decoy option was rarely chosen (0.003 ± 0.016).

**Figure 2:**
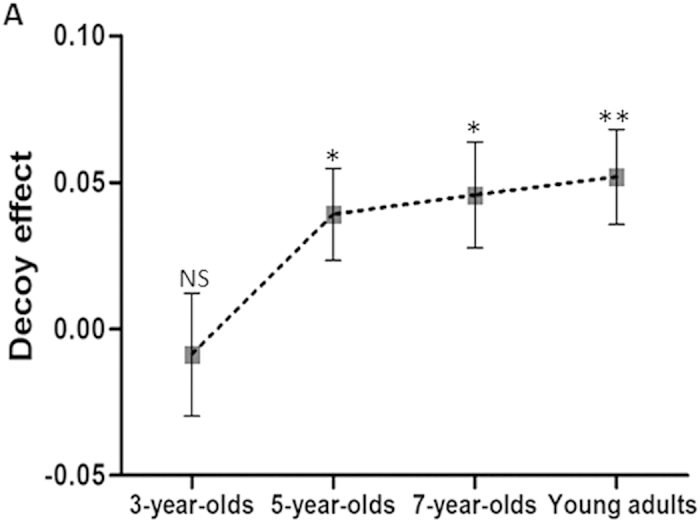
Behavioral results. (**A**) Mean decoy effect (the proportion of choosing the target option minus the proportion of choosing the competitor option) as a function of age group (3-year-olds, 5-year-olds, 7-year-olds and young adults). Error bars represent standard errors. NS, not significant. *p < 0.05. **p < 0.01.

As previous studies presented an index of the decoy effect for each participant^12^,^13^, here, the decoy effect was the proportion of choosing the target option minus the proportion of choosing the competitor option. First, for each child group, we conducted a one-sample t-test against a test mean of 0 and found a prominent decoy effect in both 7-year-olds (*t*(52) = 2.527, *p* = 0.015, *d* = 0.347) and 5-year-olds (*t*(53) = 2.483, *p* = 0.016, *d* = 0.338; see Fig. 2A) while 3-year-olds’ decoy effect did not differ significantly from 0 (*p* = 0.678), meaning that a significant decoy effect emerged at the 5-year-old group and 7-year-old group.

Second, we conducted a one-way ANOVA to analyze the age difference in children’s decoy effect, using the age group as the between-subject variable, and the proportion of the individual’s decoy effect as a dependent variable. There was a marginally significant main effect of age (*F*(2, 158) = 2.619, *p* = 0.076, ηp^2^ = 0.032; Fig. 2A). Post-hoc analysis found a marginally significant difference between the decoy effect of 7-year-olds (0.046 ± 0.018) and 3-year-olds (−0.009 ± 0.021; *p* = 0.094). No significant difference was observed between the other age groups (*p* > 0.150).

To further examine an individual’s preference for different types of rectangle (Height rectangle vs. Width rectangle), we compared the choice proportion of the Width rectangle with the Height rectangle across all trials in each child group (i.e., ((W target + W competitor) – (H target + H competitor))/2), using a one-sample t-test. We observed a significant preference for the Width rectangle over the Height rectangle in each group (3-year-olds: *t*(53) = 8.576, *p* < 0.001, *d* = 1.167; 5-year-olds: *t*(53) = 4.028, *p* < 0.001, *d* = 0.548; 7-year-olds: *t*(52) = 6.331, *p* < 0.001, *d* = 0.870). However, for each child group, there was no significant correlation between individuals’ preference for different types of rectangle and their own decoy effect (3-year-olds: r = −0.012, *p* = 0.932; 5-year-olds: r = 0.189, *p* = 0.170; 7-year-olds: r = 0.078, *p* = 0.579), suggesting that the decoy effect in each group was not modulated by their preference for different types of rectangle.

For the young adult group, a one-sample t-test also found a significant decoy effect (*t*(51) = 3.193, *p* = 0.002, *d* = 0.443; Fig. 2A). However, neither a significant preference for different type of rectangle (*t*(51) = 0.745, *p* = 0.460, *d* = 0.103) nor a significant correlation between individuals’ preference for different types of rectangle and their decoy effect was found (r = 0.168, *p* = 0.233). In addition, when using a one-way ANOVA to analyze the decoy effect among 5-year-olds, 7-year-olds and young adults, we observed no difference among these three groups (*p* = 0.864), suggesting that 5- to 7-year-old children already exhibited the decoy effect comparable to the level of adults.

Finally, the time limit for responding was long (i.e., 15 s) in our game, but the mean reaction time for each group was shorter than 15 s. For young adults, the mean reaction time was less than 2 s (target: 1293.462 ± 73.247 ms, competitor: 1279.921 ± 75.742 ms); for 3-year-olds, the mean reaction time was less than 6 s (target: 4902.085 ± 190.908 ms, competitor: 5451.663 ± 655.33 ms); for 5-year-olds, the mean reaction time was less than 5 s (target: 4247.419 ± 162.313 ms, competitor: 4214.033 ± 157.8573 ms); for 7-year-olds, the mean reaction time was less than 4 s (target: 3551.216 ± 187.756 ms, competitor: 3452.649 ± 191.147 ms). There was no significant difference between the reaction time of choosing the target and competitor for each group (*p* > 0.1). However, this response time data may not reflect children’s psychological processes for their choice, because the key pressing was made by the experimenter and may have taken variable extra time for each child.

## Discussion

This experiment investigated how the decoy effect develops in young children. Our results showed that older children (5- to 7-year-old) were sensitive to the decision context, with preferences for the target options being significantly increased in the presence of a decoy option in the age 5 and 7 groups. Meanwhile, 3-year-old children’s preferences were less affected by the decoy option, suggesting that younger children do not show a significant decoy effect in making choices. Our study indicates that the decoy effect gradually develops with age and emerges explicitly at 5- to 7-years.

To our knowledge, our study is the first to demonstrate a developmental trend of context-dependent preferences of multi-alternative choices in young children. One aim of this study was to determine whether the decoy effect is learned from social experience. Evidently, our results support the notion that children need more social experience to show significant context-dependent preferences in decision making, even in a simple perceptual discrimination task. It could be reasoned that 3-year-olds might have less experience than older children with using contextual information (decoy option) to help them make decisions. Some studies have provided evidence that 4-year-old children classify different objects depending on the objects’ irrelevant label rather than their perceptual similarity^23^,^27^,^28^, suggesting that the use of contextual information in decision-making is not an inherent strategy in younger children. Furthermore, some researchers have shown that heuristics are adopted in social learning, which increases an individual’s adaptation to cultural trends and helps develop social intelligence^29^,^30^,^31^,^32^.

Thus, our findings may support the view that the decoy effect is an environmental process, because children above the age of 5 have more social experience in decision making and they tend to use the decoy option as a cue more often than 3-year-olds. In addition, just as our hypothesis that young children’s weaker cognitive ability may increase their decoy effect in such a context-dependent choice set, cognitive ability could strongly influence children’s judgments in other dimensions as well. For instance, one study showed that children before 6 or 7 years of age cannot consistently compare relative values of judgments and their real motivations in an economic game, which may result from limited cognitive capacity^33^. Similarly, comparing relative values between decoy and target options in our perceptual task may require an understanding of a comparative calculation and proportionality^33^. However, young children’s understanding of probability events is a late developing ability^34^. For example, children as young as age 5 showed some understanding of probability dependence, whereas children at and above the age of 8 understand the idea that probability should act as a multiplier of the value in assessing the desirability of various gambles^35^,^36^. Thus, the fact that 3-year-old children show no significant decoy effect may be due to their limited cognitive ability. It is worth noting that such cognitive ability would improve with the development of social experience and learning skills, which would lead to an increasing of decoy effect with age.

Notably, as previously pointed out, animal studies of the decoy effect suggest it may have an evolutionary basis, and many innate processes emerge later in development (e.g., puberty). The fact that younger children did not show the decoy effect does not necessarily mean it is learned. Thus, one alternative explanation is that the decoy effect only emerges at a certain age based on genetic regulation. Past research has found that older adults showed no decoy effect across a range of topic domains, suggesting that increasing experience with decisions may result in skilled decision making that is independent of interest level or knowledge about a domain^37^,^38^. It is possible that older adults may have more experience in overcoming heuristics in their life experience, making them less vulnerable to the decoy effect. These studies suggest that experience is crucial for the use of heuristics such as this one. Taken together, our results indicate that people’s comparative evaluation strategy in decision making (i.e., using the decoy option to compare the dimensional values of the target on one dimension relative to the competitor) may require the use of social experience and the development of cognitive ability. This means that humans need to learn not to evaluate options separately, but rather to evaluate them by their relative value^8^,^18^,^19^,^20^. It seems that both the experience of using heuristics and the experience of overcoming heuristics influence individuals’ decision strategies.

There are a few potential limitations in this study worth mentioning. First, we only recruited Han Chinese children and adults, and as our perceptions are rooted in culture, it is unclear whether our findings would remain the same for children from Western cultures. It is well established that Asians tend to engage in context dependent and holistic perceptual processes by attending to the relation between the object and the context in which the object is located, whereas Westerners tend to engage in context-independent and analytic perceptual processes by focusing on a salient object independent of its context^39^. It is possible that the development of a decoy effect might be delayed in Western children, compared with Asian children. Future research may apply a cross-culture approach to further investigate this issue.

Second, we only investigated the contextual effect in situations where contextual information should be ignored. It would be interesting to examine contextual effects in situations where it is beneficial to take contextual information into account. Third, the contextual effects also include other effects. These include the similarity effect in which the preference for a dissimilar option would be increased by adding a similar option to the original choice set^40^, the compromise effect in which an option becomes more attractive when it is presented as a compromise between members of the original choice set^15^, and the phantom decoy effect in which an alternative that is superior to another “target” option, but is unavailable at the time of choice. Whether our current findings can be extended to other forms of contextual effects remains to be tested.

Fourth, our study only examined the decoy effect in the perceptual domain. It is unclear how the decoy effect in the perceptual domain relates to the decoy effect in other types of domains. Whether the same pattern can be found using a non-perceptual task remains to be tested. Fifth, the older children in our sample exhibited a decoy effect that was not significantly different from that of the young adults in our perceptual paradigm, suggesting that children as young as age 5 could have a sense of context information integration similar to that of adults. Another possibility is that our task is too easy to tap high level comparative evaluation strategies, so that the decoy effect in our study showed no significant difference between 5-year-olds and young adults. Future studies may develop more sophisticated paradigms to test this hypothesis. The finding that young children exhibited a significant preference for the Width rectangle over the Height rectangle is interesting and worth further investigation. Finally, our study is silent about the exact strategies/heuristics older children use in making context-dependent choices. Future studies may further examine the exact psychological processes underlying the decoy effect in children.

Understanding the decoy effect has strong implications for personal, financial, and social well-being. Our results suggest that the asymmetrically dominated decoy effect is not inherent but develops at a later age in young children, at around age 5. This raises an important question of how the decoy effect is linked to cognitive development and social experience.

## Additional Information

**How to cite this article**: Zhen, S. and Yu, R. The development of the asymmetrically dominated decoy effect in young children. *Sci. Rep.* **6**, 22678; doi: 10.1038/srep22678 (2016).

## Figures and Tables

**Table 1:** The height and width (in pixels) of the rectangles used in each game trial.

Target type	Target		Decoy		Competitor	
	Width	Height	Width	Height	Width	Height
Width target	77	41	77	33	41	77
	77	41	77	32	41	77
	77	46	77	38	46	77
	77	46	77	37	46	77
	77	51	77	43	51	77
	77	51	77	42	51	77
	82	41	82	33	41	82
	82	41	82	32	41	82
	82	46	82	38	46	82
	82	46	82	37	46	82
	82	51	82	43	51	82
	82	51	82	42	51	82
	87	41	87	33	41	87
	87	41	87	32	41	87
	87	46	87	38	46	87
	87	46	87	37	46	87
	87	51	87	43	51	87
	87	51	87	42	51	87
Height target	41	77	33	77	77	41
	41	77	32	77	77	41
	41	82	33	82	82	41
	41	82	32	82	82	41
	41	87	33	87	87	41
	41	87	32	87	87	41
	46	77	38	77	77	46
	46	77	37	77	77	46
	46	82	38	82	82	46
	46	82	37	82	82	46
	46	87	38	87	87	46
	46	87	37	87	87	46
	51	77	43	77	77	51
	51	77	42	77	77	51
	51	82	43	82	82	51
	51	82	42	82	82	51
	51	87	43	87	87	51
	51	87	42	87	87	51

Note. Width target was one in which the height of the target option was greater than width, whereas height target was one in which the width of the target option was greater than height.

## References

[b1] Arrow K. J. Risk perception in psychology and economics. Econ. Inquiry 20, 1–9 (1982).

[b2] Slaughter J. E., Kausel E. E. & Quiñones M. A. The decoy effect as a covert influence tactic. J. Behav. Decis. Making 24, 249–266 (2011).

[b3] Einhorn H. J. Expert judgment: Some necessary conditions and an example. J. Appl. Psychol. 59, 562 (1974).

[b4] Gaeth G. J. & Shanteau J. Reducing the influence of irrelevant information on experienced decision makers. Organ. Behav. Hum. Perf. 33, 263–282 (1984).

[b5] Sullivan K. & Kida T. The effect of multiple reference points and prior gains and losses on managers′ risky decision making. Organ. Behav. Hum. Dec. 64, 76–83 (1995).

[b6] Huber J., Payne J. W. & Puto C. Adding asymmetrically dominated alternatives: Violations of regularity and the similarity hypothesis. J. Cons. Res. 9, 90–98 (1982).

[b7] Ray P. Independence of irrelevant alternatives. Econometrica: Journal of the Econometric Society 41, 987–991 (1973).

[b8] Tversky A. Intransitivity of preferences. Psychol. Rev. 76, 31 (1969).

[b9] Ariely D. & Wallsten T. S. Seeking subjective dominance in multidimensional space: An explanation of the asymmetric dominance effect. Organ. Behav. Hum. Dec. 63, 223–232 (1995).

[b10] Choplin J. M. & Hummel J. E. Comparison-induced decoy effects. Mem. Cognition 33, 332–343 (2005). 10.3758/bf03195321 16028587

[b11] Pettibone J. C. & Wedell D. H. Examining models of nondominated decoy effects across judgment and choice. Organ. Behav. Hum. Dec. 81, 300–328 (2000). 10.1006/obhd.1999.2880 10706818

[b12] Pettibone J. C. & Wedell D. H. Testing alternative explanations of phantom decoy effects. J. Behav. Decis. Making 20, 323–341 (2007).

[b13] Hu J. & Yu R. The neural correlates of the decoy effect in decisions. Frontiers in behavioral neuroscience 8, 271 (2014). 25147516 10.3389/fnbeh.2014.00271 PMC4124704

[b14] Trueblood J. S., Brown S. D., Heathcote A. & Busemeyer J. R. Not just for consumers context effects are fundamental to decision making. Psychol. Sci. 24, 901–908 (2013). 23610134 10.1177/0956797612464241

[b15] Simonson I. Choice based on reasons: The case of attraction and compromise effects. J. Cons. Res. 16, 158–174 (1989).

[b16] Wedell D. H. Distinguishing among models of contextually induced preference reversals. J. Exp. Psychol. -Learn. Mem. Cogn. 17, 767 (1991).

[b17] Bateson M., Healy S. D. & Hurly T. A. Irrational choices in hummingbird foraging behaviour. Anim. Behav. 63, 587–596 (2002).

[b18] Bateson M., Healy S. D. & Hurly T. A. Context–dependent foraging decisions in rufous hummingbirds. P. Roy. Soc. Lond. B. Bio. 270, 1271–1276 (2003). 10.1098/rspb.2003.2365 PMC1691372 12816640

[b19] Shafir S., Waite T. A. & Smith B. H. Context-dependent violations of rational choice in honeybees (Apis mellifera) and gray jays (Perisoreus canadensis). Behav. Ecol. Sociobiol. 51, 180–187 (2002).

[b20] Shafir S. Intransitivity of preferences in honey bees: support for ‘comparative’ evaluation of foraging options. Anim. Behav. 48, 55–67 (1994).

[b21] Parrish A. E., Evans T. A. & Beran M. J. Rhesus macaques (Macaca mulatta) exhibit the decoy effect in a perceptual discrimination task. Attention, Perception, & Psychophysics 77, 1715–1725 (2015). 10.3758/s13414-015-0885-6 PMC4470728 25832189

[b22] Dhar R. & Glazer R. Similarity in context: Cognitive representation and violation of preference and perceptual invariance in consumer choice. Organ. Behav. Hum. Dec. 67, 280–293 (1996).

[b23] Gelman S. A. & Markman E. M. Categories and induction in young children. Cognition 23, 183–209 (1986). 3791915 10.1016/0010-0277(86)90034-x

[b24] Josiam B. M. & Hobson J. P. Consumer choice in context: the decoy effect in travel and tourism. Journal of Travel Research 34, 45–50 (1995).

[b25] O’Curry Y. P. S. & Pitts R. The attraction effect and political choice in two elections. J. Consum. Psychol. 4, 85–101 (1995).

[b26] Ebeling K. S. & Gelman S. A. Children’s use of context in interpreting “big” and “little”. Child Dev. 65, 1178–1192 (1994). 7956473

[b27] Jones S. S. & Smith L. B. The place of perception in children’s concepts. Cognitive Development 8, 113–139 (1993).

[b28] Jaswal V. K. & Markman E. M. Looks aren’t everything: 24-month-olds’ willingness to accept unexpected labels. J. Cogn. Dev. 8, 93–111 (2007).

[b29] Laland K. N. Social learning strategies. Anim. Learn. Behav. 32, 4–14 (2004). 10.3758/bf03196002 15161136

[b30] Boyd R. & Richerson P. J. Culture and the Evolutionary Process (ed. Walters S. D. ) 172–202 (University of Chicago Press, 1988).

[b31] Whiten A. & Van Schaik C. P. The evolution of animal ‘cultures’ and social intelligence. Phil. Trans. R. Soc. B 362, 603–620 (2007). 17255007 10.1098/rstb.2006.1998 PMC2346520

[b32] Wood L. A., Kendal R. L. & Flynn E. G. Context-dependent model-based biases in cultural transmission: children’s imitation is affected by model age over model knowledge state. Evol. Hum. Behav. 33, 387–394 (2012).

[b33] Benenson J. F. *et al.* Do Young Children Understand Relative Value Comparisons ? Plos One 10, 4 (2015). 10.1371/journal.pone.0122215 PMC4398488 25875949

[b34] Hook J. & Cook T. D. Equity theory and the cognitive ability of children. Psychol. Bull. 86, 429 (1979).

[b35] Harbaugh W. T., Krause K. & Vesterlund L. Risk attitudes of children and adults: Choices over small and large probability gains and losses. Exper. Econ. 5, 53–84 (2002).

[b36] Schlottmann A. & Anderson N. H. Children’s judgments of expected value. Dev. Psychol. 30, 56 (1994).

[b37] Kim S. & Hasher L. The attraction effect in decision making: Superior performance by older adults. The Quarterly Journal of Experimental Psychology Section A 58, 120–133 (2005). 10.1080/02724980443000160 PMC1751469 15881294

[b38] Tentori K., Osherson D., Hasher L. & May C. Wisdom and aging: Irrational preferences in college students but not older adults. Cognition 81, B87–B96 (2001). 11483173 10.1016/s0010-0277(01)00137-8

[b39] Nisbett R. E. & Miyamoto Y. The influence of culture: holistic versus analytic perception. Trends Cogn. Sci. 9, 467–473 (2005). 16129648 10.1016/j.tics.2005.08.004

[b40] Tversky A. Elimination by aspects: A theory of choice. Psychol. Rev. 79, 281 (1972).

